# Flexible HIV-1 Biosensor Based on the Au/MoS_2_ Nanoparticles/Au Nanolayer on the PET Substrate

**DOI:** 10.3390/nano9081076

**Published:** 2019-07-26

**Authors:** Minkyu Shin, Jinho Yoon, Chanyong Yi, Taek Lee, Jeong-Woo Choi

**Affiliations:** 1 Department of Chemical & Biomolecular Engineering, Sogang University, 35 Baekbeom-Ro, Mapo-Gu, Seoul 04107, Korea; 2 Department of Chemical Engineering, Kwangwoon University, Wolgye-dong, Nowon-gu, Seoul 01899, Korea

**Keywords:** nanolayer, flexible biosensor, HIV-1, MoS_2_ nanoparticles, PET substrate, gp120

## Abstract

An electrochemical flexible biosensor composed of gold (Au), molybdenum disulfide nanoparticles (MoS_2_ NPs), and Au (Au/MoS_2_/Au nanolayer) on the polyethylene terephthalate (PET) substrate is developed to detect envelope glycoprotein GP120 (gp120), the surface protein of HIV-1. To fabricate the nanolayer on the PET substrate, Au is sputter coated on the flexible PET substrate and MoS_2_ NPs are spin coated on Au, which is sputter coated once again with Au. The gp120 antibody is then immobilized on this flexible electrode through cysteamine (Cys) modified on the surface of the Au/MoS_2_/Au nanolayer. Fabrication of the biosensor is verified by atomic force microscopy, scanning electron microscopy, and cyclic voltammetry. A flexibility test is done using a micro-fatigue tester. Detection of the gp120 is measured by square wave voltammetry. The results indicate that the prepared biosensor detects 0.1 pg/mL of gp120, which is comparable with previously reported gp120 biosensors prepared even without flexibility. Therefore, the proposed biosensor supports the development of a nanomaterial-based flexible sensing platform for highly sensitive biosensors with flexibility for wearable device application.

## 1. Introduction

Flexible biosensors composed of polymer materials have recently attracted significant attention for their application in wearable devices and point-of-care (POC) diagnostic systems. Polymer substrates such as polyethylene terephthalate (PET), polyimide (PI), polycarbonate (PC), and polydimethylsiloxane (PDMS) are widely used as flexible substrates [1,2]. In addition, in order to fabricate flexible biosensors, various types of nanomaterials such gold nanoparticles (GNPs), carbon nanotubes (CNTs), and graphene oxide (GO) have been introduced for granting the conductivity, enhancing the electron transfer, and biocompatibility [3,4,5,6,7,8]. Among these nanomaterials, carbon-based nanomaterials have been widely used due to their exceptional properties such as excellent electrical conductivity, high specific activated surface area, and chemical/biological stability [9,10]. 

Recently, transition metal dichalcogenide (TMD) materials such as tungsten diselenide (WSe_2_) and molybdenum disulfide (MoS_2_) have been widely researched for their application in biosensors due to unique properties including electric charge effect and semiconducting property [11,12,13]. In particular, MoS_2_ has many advantages for biosensor development due to its electrochemical property and biocompatibility [14,15]. In addition, in order to maximize the benefits of MoS_2_ on a large activated surface area, MoS_2_ nanoparticles (MoS_2_ NPs) have recently been synthesized and applied to develop various sensors [16]. However, most studies related to flexible biosensors composed of nanomaterials have been done through complex manufacturing methods. To replace the complex manufacturing methods with a simple method for flexible biosensor fabrication, our group reported the flexible biosensor fabricated by a simple manufacturing method using the sputter coating and spin coating on a PI polymer substrate for uniform nanolayer formation. To fabricate an excellent flexible biosensor, PET can be an excellent candidate as the flexible substrate due to its properties including low cost, excellent thermal conductivity, chemical resistance, and dimensionally stability compared with previously used PI substrate [17,18]. 

Numerous diseases including influenza, severe acute respiratory syndrome (SARS), and acquired immunodeficiency syndrome (AIDS) are caused by small infectious agents called viruses [19]. Among the various lethal viruses, human immunodeficiency virus (HIV) has received massive attention since it causes AIDS which aggravates the human immune system, eventually resulting in death. Since HIV has a long incubation period and can be transmitted during this incubation period, its early and accurate diagnosis is a highly critical issue in the biomedical field [20,21]. The surface of HIV is composed of an envelope glycoprotein made of Envelope glycoprotein GP120 (gp120) and glycoprotein GP41 (gp41) which are connected non-covalently. The gp120 binds with the cluster of differentiation 4 (CD4) receptors and thus, plays an important role in HIV infection [22,23]. Until now, to accurately detect HIV, various methods such as enzyme-linked immunosorbent assay (ELISA) and polymerase chain reaction (PCR) have been used [24,25]. However, these methods have limitations such as complex sample treatment process, time consumption, and low sensitivity [26]. To overcome these limitations, electrochemical immunosensors have been developed for high sensitivity and real-time detection of HIV.

From these points of view, MoS_2_ nanoparticles (NPs), PET and gp120 can be the core components to develop the HIV biosensor with flexibility and high sensitivity. In this study, an electrochemical flexible biosensor based on gold (Au), MoS_2_ NPs, and Au (Au/MoS_2_/Au nanolayer) on a PET substrate was fabricated for the first time to detect the HIV surface protein gp120 using poly(vinylpyrrolidone) (PVP)-modified MoS_2_ NPs on the PET substrate. PVP-modified MoS_2_ NPs were synthesized to prevent aggregation of MoS_2_ NPs, and to facilitate their effective dispersion on the PET substrate. Synthesis of PVP-modified MoS_2_ NPs was verified by transmission electron microscopy (TEM), energy-dispersive X-ray spectroscopy (EDS), and X-ray diffraction (XRD), while the fabrication of the Au/MoS_2_/Au nanolayer on the PET substrate was confirmed by field emission scanning electron microscopy (FE-SEM), EDS, and atomic force microscopy (AFM). A gp120 antibody (Ab) was then immobilized on the substrate through the 1-Ethyl-3-(3-dimethylaminopropyl) carbodiimide (EDC)/ N-hydroxysuccinimide (NHS) reaction in order to prepare the biosensor (Ab/Cys/Au/MoS_2_/Au nanolayer). Immobilization of the gp120 antibody was confirmed by cyclic voltammetry (CV). In addition, the electrochemical property for gp120 detection was confirmed by CV and square wave voltammetry (SWV). A micro-fatigue tester (E3000LT, Instron, Buckinghamshire, UK.) was used to evaluate the flexibility of the fabricated Au/MoS_2_/Au nanolayer on the PET substrate. 

## 2. Materials and Methods 

### 2.1. Materials

Ammonium molybdate tetrahydrate (99.98%, Sigma-Aldrich, Burlington, MA, USA), thiourea (AMRESCO, Solon, OH, USA), and polyvinylpyrrolidone (Sigma-Aldrich, Burlington, MA, USA) were used to synthesize the MoS_2_ NPs. Potassium hexacyanoferrate (III) (K_3_Fe(CN)_6_) (approx. 99.0%, Sigma-Aldrich, Burlington, MA, USA) and potassium hexacyanoferrate (II) trihydrate (K_4_Fe(CN)_6_) (≥99.0%, Sigma-Aldrich, Burlington, MA, USA) solution in phosphate-buffered saline (PBS) (Sigma-Aldrich, Burlington, MA, USA) was used as the electrolyte in this study. EDC and NHS were purchased from Thermo Scientific (≥99.0%, Waltham, MA, USA). Human serum (Sigma-Aldrich, Burlington, MA, USA), cysteamine (Cys) (≥98.0%, Sigma-Aldrich, Burlington, MA, USA), gp120 antibody (Sino biological, Wayne, PA, USA), and gp120 antigen (ACRO Biosystem, Newark, DE, USA) were used to fabricate the biosensor. All aqueous solutions were prepared using deionized (DI) water from a Millipore Milli-Q water purifier operating at a resistance of 18 MΩ·cm. Myoglobin (Mb) (≥90.0%, Sigma-Aldrich, Burlington, MA, USA), hemoglobin (Hb) (Sigma-Aldrich, Burlington, MA, USA), thioredoxin (Trx) (Sino biological, Wayne, PA, USA), and prostate-specific antigen (PSA) (Abcam, Cambridge, UK) were used to investigate the selectivity of the fabricated biosensor.

### 2.2. MoS_2_ NPs Synthesis 

Ammonium molybdate tetrahydrate (0.35 g) and 0.76 g of thiourea were mixed with 50 mL of DI water. Then 0.25 g of PVP was added to the mixture. The resulting solution was reacted at 800 rpm for 1 h at 60 °C. The solution was transferred into a Teflon-lined stainless-steel autoclave. The autoclave was set at 200 °C for 24 h. After the reaction was complete, the resulting black precipitate was cooled down to room temperature and washed with DI water and ethanol using centrifugal filtration with 8000 rpm for 30 min. The final product was air-dried at 60 °C for 24 h. The PVP-modified MoS_2_ NPs (concentration: 1 mg/mL) were characterized by high-resolution TEM using a JEOL JEM-3010 operated at 300 kV and by XRD (Rigaku, Tokyo, Japan). 

### 2.3. Fabrication of the Au/MoS_2_/Au Nanolayer on the PET Substrate, and Immobilization of the gp120 Antibody

**Figure 1:**
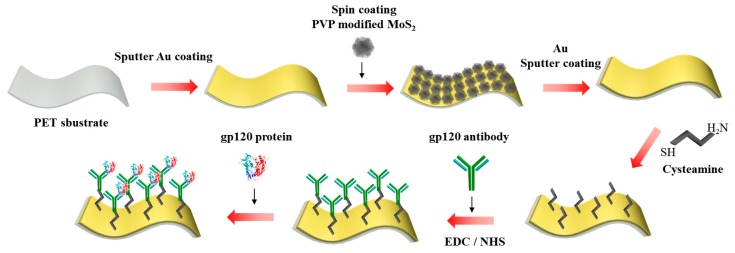
Schematic image of the fabrication of the flexible biosensor composed of gp120 antibody (Ab)/cysteamine (Cys)/Au/MoS_2_/Au nanolayer on the polyethylene terephthalate (PET) substrate for the detection of gp120.

The fabrication of a flexible biosensor composed of Ab/Cys/Au/MoS_2_/Au nanolayer on PET substrate is shown in Figure 1. To fabricate the Au/MoS_2_/Au nanolayer on the PET substrate, the rectangular PET substrate (size 1 mm × 2 mm) was cleaned in a sonication bath for 30 min using acetone and DI water, and then completely dried with N_2_ gas. After cleaning the PET, the Au/MoS_2_/Au nanolayer on the PET substrate was prepared by gold sputter and spin coater. The Au layer was formed by sputter coating on the PET substrate. Then, 200 μL of the synthesized MoS_2_ NPs dissolved in DI water (concentration: 5 mg/mL) was dropped onto the Au layer and spin coated (Au/MoS_2_) on the PET substrate at 2000 rpm for 30 s; this process was done twice. After the spin coating process, the Au layer was sputter coated once more on the MoS_2_ layer. Fabrication of the Au/MoS_2_/Au nanolayer on the PET substrate was verified by FE-SEM, EDS, and AFM. To fabricate the Ab/Cys/Au/MoS_2_/Au nanolayer on the PET substrate, the gp120 antibody was immobilized on the Au/MoS_2_/Au nanolayer on the PET substrate. The fabricated electrode was washed with ethanol and DI water in preparation for the gp120 antibody immobilization. Cys (77 mg) dissolved in 10 mL of DI water (10 mM concentration) was then immobilized on the electrode by self-assembly for 3 h at room temperature. The Cys-immobilized electrode was washed with DI water to remove any unbound Cys. Then, the gp120 antibody was attached to Cys using the EDC/NHS reaction. To achieve this, 100 μL of the antibody solution (concentration: 5 μg/mL) was mixed with 100 μL of EDC (concentration: 4 mg/mL) and 100 μL of NHS (concentration: 6 mg/mL) for 1 h at room temperature. Then, the modified gp120 antibody with EDC/NHS was dropped on the Cys-immobilized electrode for 2 h at room temperature. In addition, the gp120 antibody-immobilized electrode was washed with DI water to remove any unbound gp120 antibody and dried with N_2_ gas.

### 2.4. Electrochemical Properties of the Au/MoS_2_/Au Nanolayer on the PET Substrate

Electrochemical properties of the fabricated biosensor were analyzed by electrochemical analyzer CHI-660E (CH Instruments, Inc., Austin, TX, USA). CV and SWV were conducted with a three-electrode system composed of the fabricated working electrode, a platinum (Pt) wire counter electrode, and a silver/silver chloride (Ag/AgCl) reference electrode. The parameters for the CV experiment were as follows: sampling interval of 1 mV/s, the scan rate of 50 mV/s, and 1 × 10^−3^ (A/V) sensitivity. The applied voltage range was from 600 mV to −200 mV. The parameters for the SWV experiment were as follows: amplitude of 20 mV, frequency of 10 Hz, and 1 × 10^−3^ (A/V) sensitivity. The applied voltage range was from 500 mV to −200 mV. The electrochemical experiments were conducted with PBS solution (pH = 7.4) containing 5 mM K_3_Fe(CN)_6_ and 5 mM K_4_Fe(CN)_6_ which were used as the redox generator for electrochemical investigation. 

### 2.5. Flexibility Test of Fabricated Biosensor

The flexibility of the fabricated biosensor was investigated using a micro-fatigue tester. To estimate the flexibility, the electrode was fixed to the tester and the tip of the tester was moved down to apply force on the electrode. The electrode was bent by the force applied to the center of the electrode. The elongation of the electrode by the applied force was measured to investigate the flexibility of the electrode. The fabricated biosensor was compared with a widely used rigid gold coated silicon electrode. 

## 3. Results and Discussion

### 3.1. Confirmation of MoS_2_ NPs Synthesis

**Figure 2:**
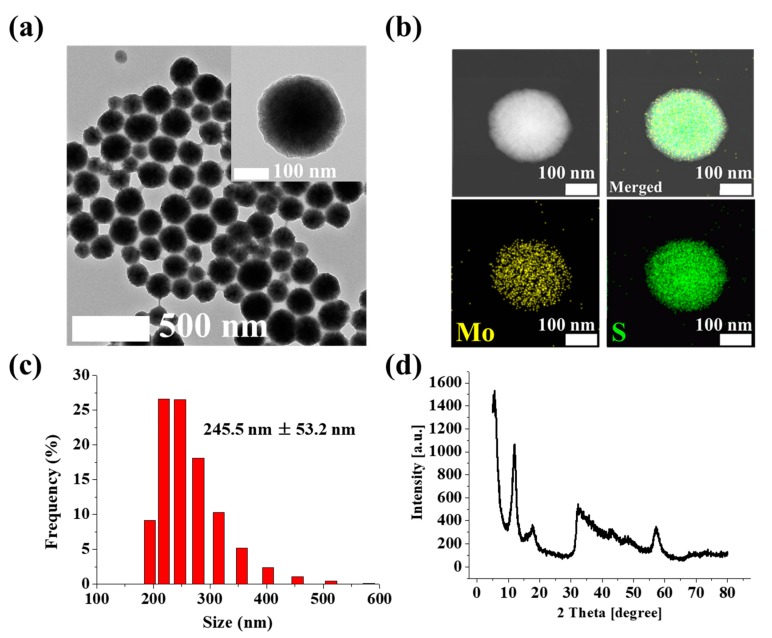
(**a**) TEM and (**b**) energy-dispersive X-ray spectroscopy (EDS) mapping images of MoS_2_ nanoparticles (NPs), (**c**) dynamic light scattering (DLS) result of MoS_2_ NPs, and (**d**) XRD pattern analysis of MoS_2_ NPs.

The conformation and composition of the synthesized MoS_2_ NPs were investigated by TEM and EDS analysis. As shown in Figure 2a, the TEM images indicated that the synthesized MoS_2_ NPs were structured as well-dispersed nanospheres. The EDS and EDS mapping results are shown in Figure 2b and Figure S1. EDS and EDS mapping images showed that the ratio of molybdenum (Mo) and sulfur (S) was approximately 1:2, which matched the theoretical composition of MoS_2_. Additionally, the average size of MoS_2_ (concentration: 1 mg/mL) was investigated by dynamic light scattering (DLS). The measured DLS data indicated that 80.37% of synthesized MoS_2_ were about 200 nm to 300 nm, and 19.63% of MoS_2_ were over 300 nm (Figure 2c), and the average diameter of MoS_2_ was 245.5 nm ± 53.2 nm. The XRD diffractometer analysis of MoS_2_ was performed at a scanning rate of 5°/min and with a 2Ɵ range from 5° to 80°. The XRD pattern of the synthesized MoS_2_ NPs showed peaks at 13°, 17°, 33°, 43°, and 58° for the hexagonal phase of MoS_2_ NPs shown in Figure 2d. Thus, these experiments verified the synthesis of MoS_2_.

### 3.2. Verification of the Au/MoS_2_/Au Nanolayer on the PET Substrate

**Figure 3:**
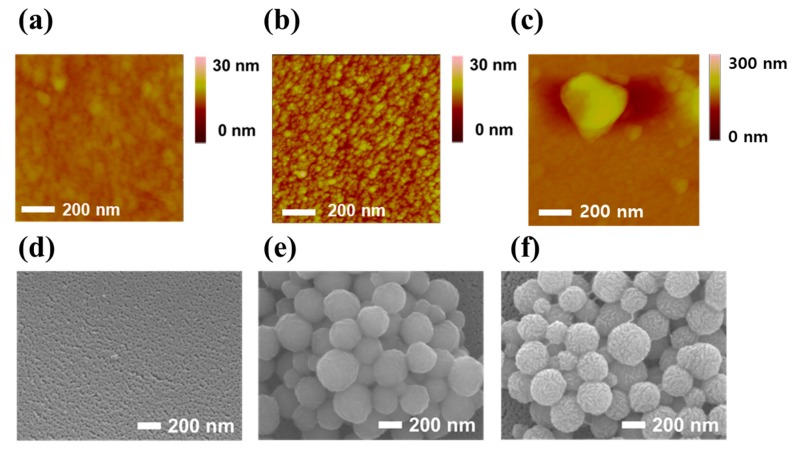
Atomic force microscopy (AFM) images of (**a**) the PET substrate, (**b**) Au sputter coated PET substrate, and (**c**) the Au/MoS_2_ on the PET substrate, and SEM images of (**d**) Au sputter coated PET substrate, (**e**) the Au/MoS_2_ on the PET substrate, and (**f**) the Au/MoS_2_/Au nanolayer on the PET substrate.

**Figure 4:**
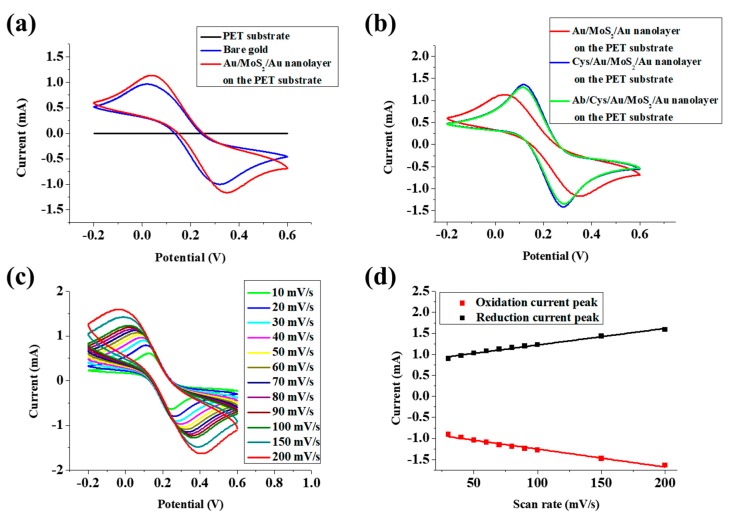
(**a**) Cyclic voltammograms of the PET substrate, bare gold, and the Au/MoS_2_/Au nanolayer on the PET substrate. (**b**) Cyclic voltammograms of the Au/MoS_2_/Au nanolayer on the PET substrate, the Cys/Au/MoS_2_/Au nanolayer on the PET substrate, and the Ab/Cys/Au/MoS_2_/Au nanolayer on the PET substrate. (**c**) Cyclic voltammograms of the Au/MoS_2_/Au nanolayer on the PET substrate at scan rates increasing from 10 to 200 mV/s. (**d**) Linear-response plot of redox current peaks of the Au/MoS_2_/Au nanolayer on the PET substrate against scan rates.

Fabrication of the Au/MoS_2_/Au nanolayer on the PET substrate was confirmed by AFM and FE-SEM. Figure 3a–c shows AFM images of the PET substrate, Au sputter coated PET substrate, and Au/MoS_2_ on the PET substrate, respectively. In Figure 3a, the AFM results of the PET substrate indicated a height of 1.296 nm. When Au was sputter coated on the PET substrate, the height of the substrate increased to 7.856 nm due to the deposited Au as shown in Figure 3b. The MoS_2_ NPs immobilized on the Au coated PET substrate by spin coating was shown in Figure 3c, the height of the immobilized MoS_2_ NPs was about 155.81 nm. In addition, Figure 3d–f shows FE-SEM images of the fabricated Au/MoS_2_/Au nanolayer on the PET substrate. Compared to the results in Figure 3e, the existence of the sputter coated Au particles on MoS_2_ NPs was confirmed in Figure 3f. EDS analysis of all the acquired SEM images is shown in Figure S2. The amount of sputter coated gold in the Au/MoS_2_/Au nanolayer on the PET substrate was doubled to the Au/MoS_2_ on the PET substrate due to the twice gold deposition. In addition, the EDS mapping result of the Au/MoS_2_/Au nanolayer on the PET substrate is shown in Figure S3. Also, the CV result is shown in Figure 4a for confirmation of the fabrication of the Au/MoS_2_/Au nanolayer on the PET substrate. In Figure 4a, the CV results of the PET substrate showed no electrochemical signals, but signals were observed after Au sputter coating on the PET substrate. Also, the fabricated Au/MoS_2_/Au nanolayer on the PET substrate had higher electrochemical signals than the conventional bare gold electrode composed of gold (50 nm)/Cr (2 nm)/SiO_2_ (Silicon dioxide). The current increase of the Au/MoS_2_/Au nanolayer on the PET substrate compared to conventional bare gold electrode was due to the large surface area and the efficient electron transfer by MoS_2_ NPs.

### 3.3. Investigation of the Electrochemical Properties of the Fabricated Biosensor

CV and SWV were performed to confirm the electrochemical properties of the fabricated biosensor with a [Fe(CN)_6_]^3−/4−^ redox probe. Figure 4b shows the CV result of the Au/MoS_2_/Au nanolayer on the PET substrate, the Cys/Au/MoS_2_/Au nanolayer on the PET substrate, the Ab/Cys/Au/MoS_2_/Au nanolayer on the PET substrate. The reduction and oxidation peak currents of the Au/MoS_2_/Au nanolayer on the PET substrate was found to be 1.13 mA and −1.17 mA, respectively. After Cys immobilization, the reduction and oxidation peak potentials were changed from 0.05 V and 0.35 V to 0.12 V and 0.28 V, and the reduction and oxidation peak potentials were also increased to 1.36 mA and −1.37 mA, respectively. This increase was due to the electrostatic interaction between the negatively charged [Fe(CN)_6_]^3−/4−^ and the positively charged amine group of the Cys immobilized on the Au/MoS_2_/Au nanolayer on the PET substrate. However, when the gp120 antibody was immobilized on the Cys, the efficiency of electron transfer between the multilayer and the redox probe was reduced by the gp120 antibody, and the reduction and oxidation peak currents were lowered to 1.30 mA and −1.34 mA, respectively. In addition, to confirm the reproducibility of fabricated biosensor, the average reduction peak current was investigated with four different measurements. As shown in Figure S4, the average reduction peaks of Au/MoS_2_/Au nanolayer on the PET substrate, Cys/Au/MoS_2_/Au nanolayer on the PET substrate and Ab/Cys/Au/MoS_2_/Au nanolayer on the PET substrate were 1.12 mA, 1.38 mA, and 1.30 mA. In Figure 4c, the CV results were obtained by increasing the scan rate from 10 mV/s to 200 mV/s to verify the relationship between the current peak and the scan rate of the fabricated the Au/MoS_2_/Au nanolayer on the PET substrate. Figure 4d showed that the plotted reduction and oxidation peak currents of the measured CV showed a linear response to the increase in the scan rate.

### 3.4. Detection of the gp120 Antigen and Selectivity

**Figure 5:**
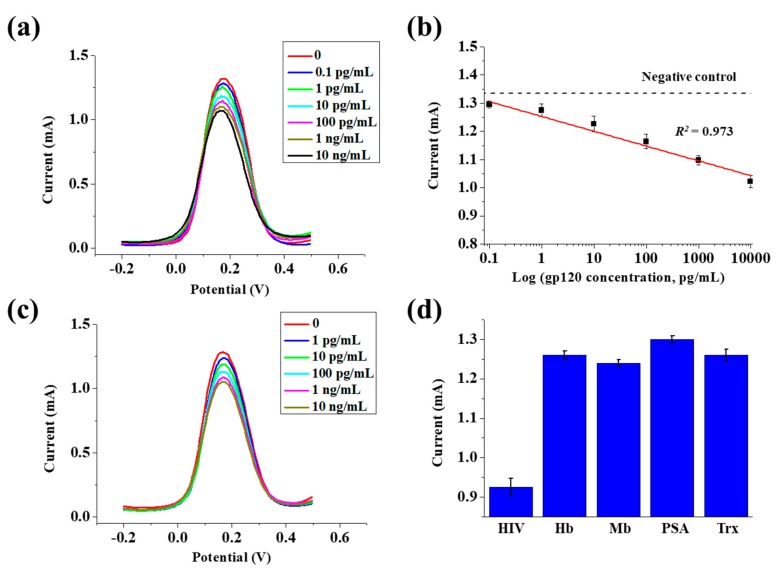
(**a**) Square wave voltammetry (SWV) results and (**b**) linear-response plot of the current peak values for the concentration of gp120 from 0.1 pg/mL to 10 ng/mL in phosphate-buffered saline (PBS) solution. (**c**) SWV results for the concentration of gp120 from 1 pg/mL to 10 ng/mL in serum. (**d**) Selectivity test of the Ab/Cys/Au/MoS_2_/Au nanolayer on the PET substrate to various types of antigens and proteins including Hb, Mb, PSA, and Trx prepared in PBS solution.

The SWV technique was performed to investigate the electrochemical detection performance and selectivity of the fabricated biosensor. To confirm its detection, the gp120 antigen dissolved in PBS solution was immobilized on the prepared Ab/Cys/Au/MoS_2_/Au nanolayer on the PET substrate for 1 h at room temperature. The detection of the gp120 antigen at a concentration of 0.1 pg/mL to 10 ng/mL was confirmed by SWV. As shown in Figure 5a, the electron transfer reaction between the redox probe and the biosensor surface was blocked by the antigen–antibody binding on the surface of the fabricated biosensor, and the current value was decreased when the concentration of the gp120 antibody was increased. In addition, the linearity of the current value for the gp120 antigen concentration was confirmed through the results obtained from SWV. As shown in Figure 5b, the gp120 antigen ranged from 0.1 pg/mL to 10 ng/mL and showed excellent linearity with 0.973 of the coefficient of determination value (*R*^2^). In general, the concentration of gp120 in HIV-infected patients is approximately 200 pg/mL to 2000 pg/mL, therefore the fabricated biosensor showed the possibility for gp120 detection in practice. The mean value and the error bars were plotted with the standard deviation (SD) from four different measurements. The detection limit of an electrochemical biosensor composed of the Au/MoS_2_/Au nanolayer on the PET substrate for HIV detection was 0.066 pg/mL based on the 3 × (*S*/*m*) method, where *S* is the standard deviation of the blank signal and *m* is slope of the linear fitting curve, and this value was compared with other electrodes shown in Table 1. To confirm the detection ability for the gp120 antigen prepared from a real sample, human serum was mixed with the gp120 antigen, and the detection of the gp120 antigen was confirmed by SWV. For real sample analysis, the gp120 with different concentrations from 1 pg/mL to 10 ng/mL was mixed with serum. As shown in Figure 5c, in the case of the gp120 antigen prepared in serum, the current value decreased with increase in concentration, the same as the gp120 antigen dissolved in PBS. The selectivity of the Ab/Cys/Au/MoS_2_/Au nanolayer on the PET substrate-based biosensor was measured by the addition of various types of antigens and proteins including Hb, Mb, PSA, and Trx prepared in PBS solution. The concentrations of the gp120 antigen, Hb, Mb, PSA, and Trx were fixed at 100 ng/mL. As shown in Figure 5d, the gp120 antigen had a low current value of 0.925 mA, while Hb, Mb, PSA, and Trx had high current values of 1.26 mA, 1.24 mA, 1.30 mA, and 1.26 mA, respectively. In addition, the rate of change of current value of gp120 antigen, Hb, Mb, PSA, and Trx were indicated 29.9%, 4.42%, 6.43%, 1.45%, and 4.60%. The mean value and the error bars were obtained as the standard deviation (SD) of five measurements. As shown in the results of the selectivity test, the measurement of gp120 was confirmed since the current value of the gp120 antigen, a surface protein of HIV was smaller than that of other antigens and proteins, and the electrochemical signal decreased due to selective immobilization of the gp120.

### 3.5. Investigation of the Flexibility of the Au/MoS_2_/Au Nanolayer on the PET Substrate

**Figure 6:**
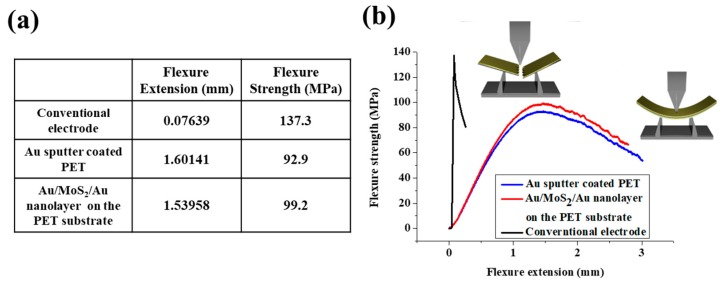
(**a**) Table of results for flexibility test and (**b**) flexure graphs of conventional electrode, Au sputter coated PET, Au/MoS_2_/Au nanolayer on the PET substrate.

The flexibility of the fabricated biosensor was confirmed by a micro-fatigue tester. Figure 6a,b show flexibility results of a conventional bare gold electrode, Au sputter coated PET substrate, and the Au/MoS_2_/Au nanolayer on the PET substrate. In Figure 6a, the flexure extension results of the conventional bare gold electrode were 0.076 mm due to the hardness of the electrode. SiO_2_-based rigid substrates were hard to bend using applied forces due to their lack of flexibility. However, Au sputter coated PET and the Au/MoS_2_/Au nanolayer on the PET substrate showed excellent flexibility and flexure extension with 1.60 mm and 1.54 mm, respectively. These results were significantly higher than those of the conventional electrode because these electrodes were easily bent by the applied force due to their excellent flexibility. In addition, the Au sputter coated PET and Au/MoS_2_/Au nanolayer on the PET substrate had flexure strength of 92.9 MPa and 99.2 MPa, respectively, which were lower than that of the conventional gold electrode due to the excellent flexibility. As shown in Figure 6b, the conventional gold electrode was found to be rapidly damaged by strong forces due to the hardness of the electrode. However, the fabricated biosensor had a high flexure extension compared with the conventional gold electrode because of the small force applied to the substrate due to the characteristics of the flexible substrate. In addition, SWV was performed to investigate electrochemical detection performance of the bent biosensor. To confirm the detection performance, 10 ng/mL of gp120 antigen was immobilized on the Ab/Cys/Au/MoS_2_/Au nanolayer on the PET substrate. As shown in Figure S5, the current value of fabricated biosensor before bent was indicated 1.06 mA, and after the fabricated biosensor was bent, the current value was maintained to 1.03 mA. 

## 4. Conclusions 

In this study, the flexible biosensor based on a Au/MoS_2_/Au nanolayer on a PET substrate was developed to detect gp120 with high sensitivity. To develop the flexible biosensor, the Au/MoS_2_/Au nanolayer was fabricated by Au sputter coating and MoS_2_ NPs spin coating onto a flexible PET substrate. The fabricated Au/MoS_2_/Au nanolayer on the PET substrate showed the well-oriented NPs and uniform nanolayer formation on the PET substrate. The reduction and oxidation peak currents of the Au/MoS_2_/Au nanolayer on the PET substrate derived from the redox generator were 1.13 mA and −1.17 mA, respectively, which were much higher than those peaks of the bare gold electrodes with 0.96 mA and −1.01 mA due to the large surface area and effective electron transfer of the synthesized MoS_2_ NPs. The fabricated biosensor showed highly sensitive detection of gp120 with a detection limit of 0.066 pg/mL, which was more sensitive than previously reported electrochemical HIV biosensors. This biosensor showed a selective detection of gp120 added with various antigens and proteins such as Hb, Mb, PSA, and Trx. In addition, this biosensor showed excellent flexibility with flexure extension of 1.54 mm compared to SiO_2_-based conventional gold electrodes, and the fabricated biosensor maintained the detection performance after bending. In conclusion, the proposed flexible biosensor based on a Au/MoS_2_/Au nanolayer on a PET substrate can suggest the milestone for nanomaterial-based flexible sensing platform to develop the highly sensitive biosensors with flexibility for a wearable device application. In addition, because the gp120 concentration range of the HIV infected patient can be measured, it can be used in the commercial field.

## Figures and Tables

**Table 1:** Electrochemical biosensors for human immunodeficiency virus (HIV) detection.

Electrode	Technique	Target	Detection Limit	Linear Range	Reference
GCE/CNF-Bi/MIP/NBD–556@ gp120	DPV	gp120	0.3 pg/mL	0.002–200 ng/mL	[27]
FP-50 fusion peptide	Dot blot	gp120	-	0–100 pg/mL	[28]
Poly(propylene imine) Dendrimer-Streptavidin Platform	SWV	gp120	4.12 pg/ml	12.04 ng/mL–0.19 μg/mL	[29]
Au nonodot/ITO	CV	HIV-1 VLP(gp120)	-	0.6–375 pg/mL	[30]
Ab/Cys/Au/MoS_2_/Au nanolayer on the PET substrate	SWV	gp120	0.066 pg/mL	0.1 pg/mL–10 ng/mL	This research

## Supplementary Materials

The following are available online at https://www.mdpi.com/2079-4991/9/8/1076/s1, Figure S1: TEM and EDS analysis of MoS_2_ NPs. Figure S2: SEM and EDS analysis of (**a**) Au sputter coated PET substrate, (**b**) the Au/MoS_2_ on the PET substrate and (**c**) the Au/MoS_2_/Au nanolayer on the PET substrate. Figure S3: EDS mapping results of the Au/MoS_2_/Au nanolayer on the PET substrate. Figure S4: The reproducibility of reduction peaks of the Au/MoS_2_/Au nanolayer on the PET substrate, Cys/Au/MoS_2_/Au nanolayer on the PET substrate and Ab/Cys/Au/MoS_2_/Au nanolayer on the PET substrate. Error bars indicate the standard deviations of four different measurements. Figure S5: Reduction peak currents of the 10 ng/mL of gp120 antigen on the Ab/Cys/Au/MoS_2_/Au nanolayer on the PET substrate before bending and after bending. Error bars indicate the standard deviations of four different measurements.
