# Supplementary material for: Flexible HIV-1 Biosensor Based on the Au/MoS2 Nanoparticles/Au Nanolayer on the PET Substrate
Source: Nanomaterials (Basel). 2019 Jul 26;9(8):1076. doi: 10.3390/nano9081076 (PMC6723525; doi:10.3390/nano9081076)
Supplement: Supplementary file 1 [file nanomaterials-09-01076-s001.pdf]

nanomaterials

Article

Flexible HIV-1 Biosensor Based on the Au/MoS_2 Nanoparticles/Au Nanolayer on the PET Substrate

Minkyu Shin [1,†], Jinho Yoon [1,†], Chanyong Yi [1], Taek Lee [2] and Jeong-Woo Choi [1,*]

1 Department of Chemical & Biomolecular Engineering, Sogang University, 35 Baekbeom-Ro, Mapo-Gu, Seoul 04107, Korea
2 Department of Chemical Engineering, Kwangwoon University, Wolgye-dong, Nowon-gu, Seoul 01899, Korea
* Correspondence jwchoi@sogang.ac.kr; Tel.: (+82)2-705-8480
† These authors contributed equally to this work.

Received: 9 July 2019; Accepted: 24 July 2019; Published: date

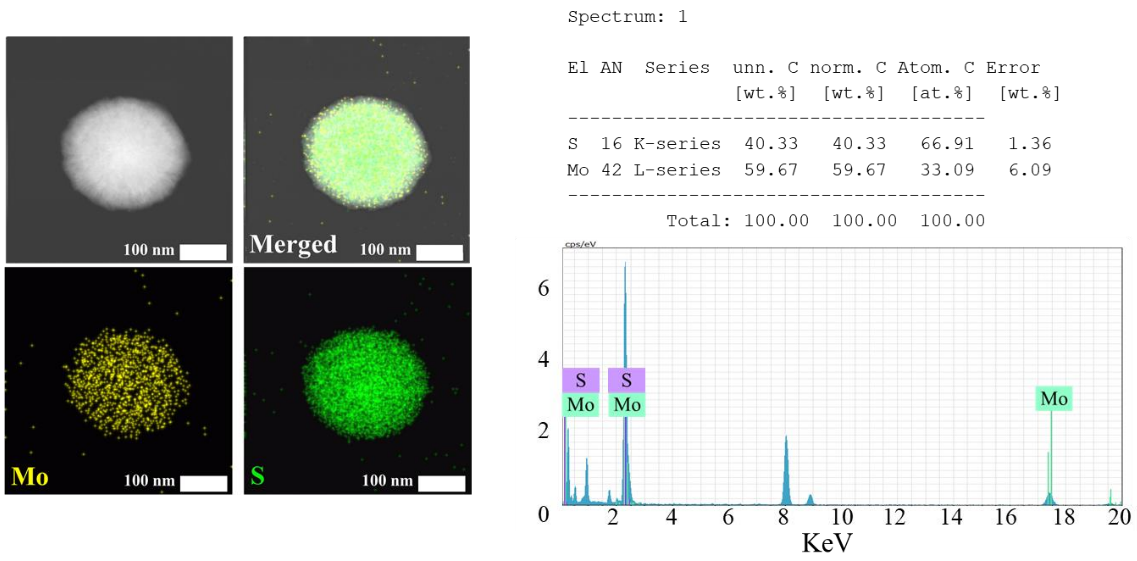

Figure S1. TEM and EDS analysis of MoS_2 NPs.

(a)

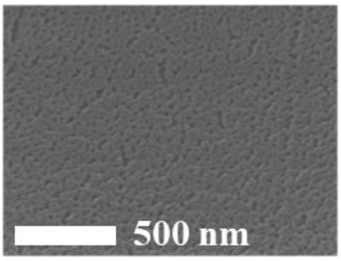

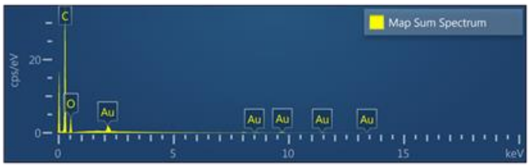

Element	Line Type	k Ratio	Wt%	Wt% Sigma	Atomic %
C	K series	0.12759	72.45	0.47	81.73
O	K series	0.00776	21.05	0.44	17.83
Au	M series	0.01687	6.49	0.27	0.45
Total:			100.00		100.00

(b)

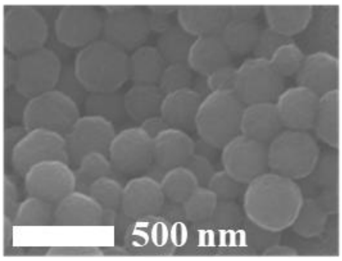

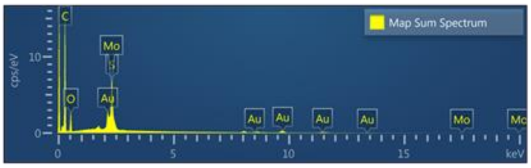

Element	Line Type	k Ratio	Wt%	Wt% Sigma	Atomic %
C	K series	0.11707	67.78	0.34	82.34
O	K series	0.01030	15.97	0.26	14.56
S	K series	0.01604	3.66	0.09	1.67
Mo	L series	0.02831	6.42	0.27	0.98
Au	M series	0.03020	6.16	0.17	0.46
Total:			100.00		100.00

(c)

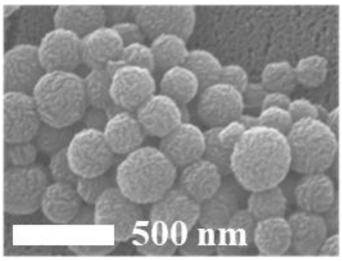

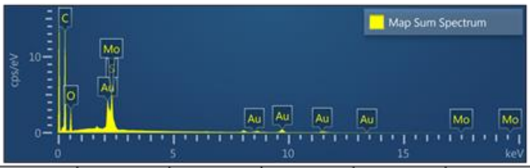

Element	Line Type	k Ratio	Wt%	Wt% Sigma	Atomic %
C	K series	0.10023	62.61	0.41	80.27
O	K series	0.01109	17.11	0.30	16.47
S	K series	0.01126	2.95	0.11	1.42
Mo	L series	0.02288	5.96	0.34	0.96
Au	M series	0.05171	11.36	0.25	0.89
Total:			100.00		100.00

Figure S2. SEM and EDS analysis of (**a**) Au sputter-coated PET substrate, (**b**) the Au/MoS_2 on the PET substrate and (**c**) the Au/MoS_2/Au nanolayer on the PET substrate.

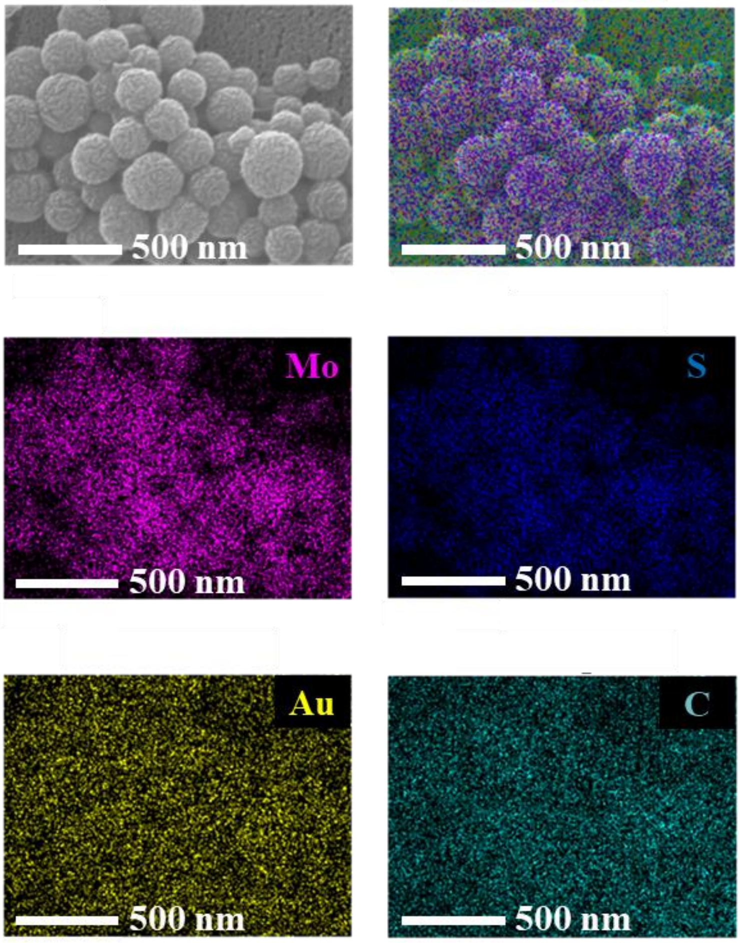

Figure S3. EDS mapping results of the Au/MoS_2/Au nanolayer on the PET substrate.

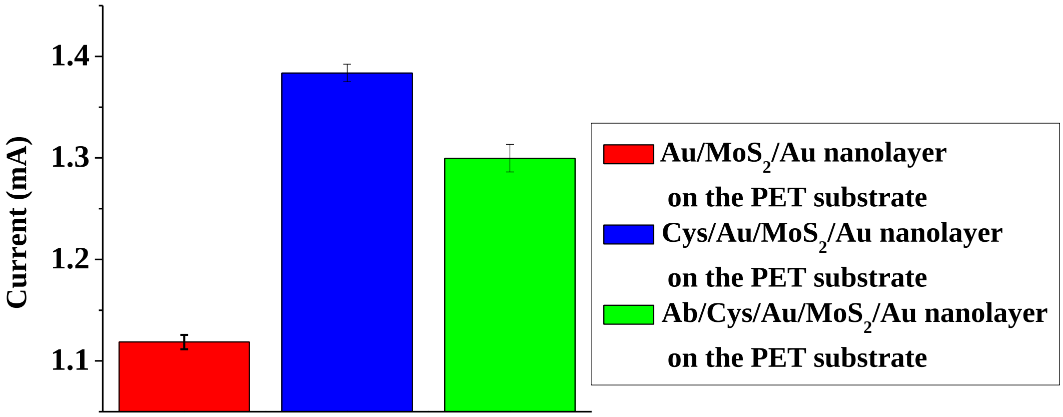

Figure S4. The reproducibility of reduction peaks of Au/MoS_2/Au nanolayer on the PET substrate, Cys/Au/MoS_2/Au nanolayer on the PET substrate and Ab/Cys/Au/MoS_2/Au nanolayer on the PET substrate. Error bars indicate the standard deviations of four different measurements.

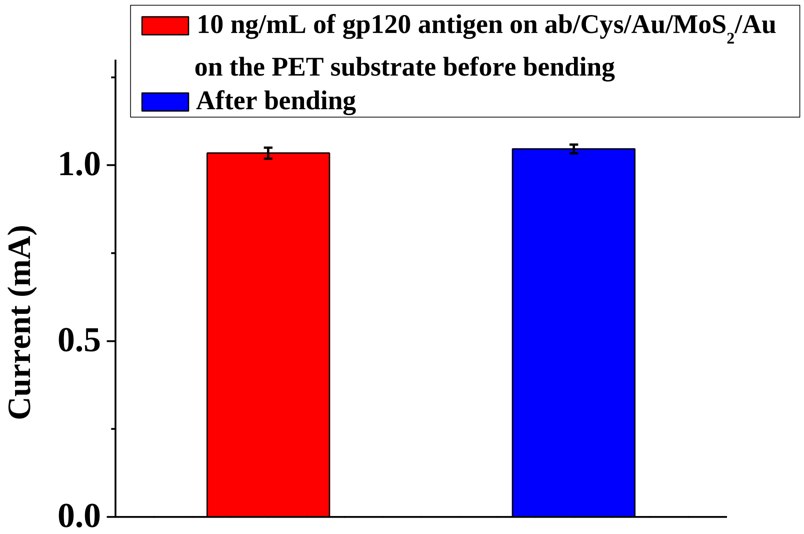

Figure S5. Reduction peak currents of the 10 ng/mL of gp120 antigen on Ab/Cys/Au/MoS_2/Au nanolayer on the PET substrate before bending and after bending. Error bars indicate the standard deviations of four different measurements.
